# Patterns of Failure in Patients With Advanced Non-Small Cell Lung Cancer Treated With Immune Checkpoint Inhibitors

**DOI:** 10.3389/fonc.2021.724722

**Published:** 2021-09-07

**Authors:** Rong Chai, Yipengchen Yin, Xuwei Cai, Xiaolong Fu, Qin Zhang

**Affiliations:** Department of Radiation Oncology, Shanghai Chest Hospital, Shanghai Jiao Tong University, Shanghai, China

**Keywords:** failure patterns, non-small cell lung cancer, immune checkpoint inhibitors, oligo-progression, consolidative radiotherapy

## Abstract

**Objective:**

The advent of immune checkpoint inhibitors (ICIs) has rapidly transformed the treatment paradigm of non-small cell lung cancer (NSCLC). Despite the durability of response to ICIs, the vast majority of patients will later develop progression. However, the failure patterns of ICI treatment are unknown. Here, our study explored the failure patterns in advanced NSCLC patients treated with ICIs.

**Methods:**

A cohort of 156 IIIB or IV NSCLC patients treated with first-/second-line ICIs were retrospectively analyzed. Patients who experienced clinical benefit and then developed progression were identified. The disease progression patterns were divided into three categories: progression in new sites, progression in existing sites, and combined progression. The number of progression sites was also recorded.

**Results:**

Before the cutoff date, 91 (77.1%) patients had experienced disease progression; 34% of patients had progressed in the last 9 months of the first year. Fifty-three (58.2%) patients had developed progression at existing lesions, and 56 (61.5%) patients had shown ≤2 progression sites (oligo-progression). In patients with oligo-progression, the median time of disease progression was 8.23 months and the counterpart (systemic progression) was 5.97 months. The oligo-progression patients showed prolonged median overall survival (27.23 months) compared with patients with systemic progression (18.87 months).

**Conclusions:**

Failure patterns of ICI therapy were predominantly “existing” sites, and the most common lesions of progression were the lung and lymph nodes. Most patients experienced oligo-progression which occurred later than systemic progression and showed prolonged overall survival. The control of the local lesions might be beneficial to improve ICI treatment efficacy.

## Introduction

Cancer immunotherapy with immune checkpoint inhibitors (ICIs) has provided dramatic changes in solid tumor treatment strategies, which utilizes host antitumor immune system to attack the tumor. The programmed cell death protein 1 (PD-1) or programmed cell death protein ligand 1 (PD-L1) blockade therapy remarkably prolongs long-term survival and improves the durable response rate in both monotherapy and combination cancer therapy. Currently, ICI therapy is approved to be used in multiple types of cancer, including non-small cell lung cancer (NSCLC), melanoma, head and neck cancer, renal cell carcinoma, and so forth. Although the response to ICIs tends to be more persistent than chemotherapy, most patients will unavoidably develop resistance.

Lung cancer, according to the International Agency for Research on Cancer, is the leading cause of cancer death and the second most diagnosed cancer (1). Particularly, NSCLC accounts for 80%~85% of all lung cancer cases. The advent of ICIs has dramatically changed the treatment landscape of NSCLC patients. It has shown unprecedented long-term, durable survival in some NSCLC patients (2–4). For the first time, the 5-year survival rate of advanced NSCLC cancer patients has been improved to 16%, a nearly three times increase from 4.7% (5). Clinical studies, such as KEYNOTE-024, CheckMate-017, and PACIFIC studies, have all shown that PD-1 or PD-L1 inhibitors can significantly improve the progression-free survival (PFS) and overall survival (OS) of NSCLC patients without epidermal growth factor receptor (EGFR) mutation/anaplastic lymphoma kinase (ALK) fusion, compared with traditional cytotoxic treatment agents (5–8).

Despite the success of ICIs, the vast majority of patients ultimately developed disease progression (9). Patterns-of-failure studies in advanced NSCLC patients treated with chemotherapy or EGFR-TKI showed that most progression occurred only at sites of disease known to exist at baseline, rather than in new sites (10–13), and these studies advanced the development of EGFR therapeutic. However, little is currently reported about the failure patterns of ICI therapy. Herein, considering the failure patterns are still unclear, we characterize the patterns of failure in patients with advanced NSCLC treated with ICIs and show subsequent therapy in these patients.

## Materials and Methods

### Patients

We retrospectively reviewed 156 patients with IIIB or IV NSCLC who received PD-1/PD-L1 inhibitor therapy in Shanghai Chest Hospital between January 2016 and December 2020. Patients were eligible if they were 18 years or older, with a performance status defined by the Eastern Cooperative Oncology Group (ECOG) between 0 and 1. All patients had received first-line or second-line anti-PD-1/PD-L1 therapy, including nivolumab, atezolizumab, durvalumab, pembrolizumab, or camrelizumab (a PD-1 inhibitor made in China). First-line ICI therapy refers to patients who had not received other systemic treatments before receiving ICI drugs. The patient has received one systemic treatment (such as chemotherapy) and then experienced disease progression, and the immunotherapy received after progression is called second-line ICI therapy. Patients enrolled had a measurable lesion for imaging evaluation according to the Response Evaluation Criteria in Solid Tumors (RECIST) guideline (version 1.1). Neoplasm staging was assessed on the basis of eighth American Joint Committee on Cancer (AJCC) staging manual. As the data were retrospective, waiver of consent was obtained.

### Study Design

We obtained the characteristic data of the patients regarding gender, age, smoking status, histology type, and the ECOG score from an inpatient medical record system. Baseline imaging with enhanced computed tomography (CT) of the chest, enhanced magnetic resonance imaging (MRI) or CT of the head, abdomen enhanced CT or B-ultrasound, whole-body bone scan (ECT), or whole-body PET/CT plus head enhanced MRI or CT was obtained for all patients before initiating immunotherapy. Follow-up scans were performed every 8 weeks until disease progression or death. All patients had been followed till the cutoff date (December 31, 2020) or death. To assess the efficacy of ICIs, we identified the patients with clinical benefit based on RECIST v1.1 criteria. Responders refer to patients in the overall group who experienced clinical benefit from ICI therapy. Clinical benefit refers to initial responses to ICI therapy: complete response (CR), partial response (PR), or stable disease (SD) for at least 6 months according to the follow-up imaging scans. PFS was recorded as the time from immunotherapy initiation until progression or death for any reason. Time to progression (TTP) was recorded as the time from immunotherapy initiation until disease progression, whereas OS was measured as the time from immunotherapy initiation to the time of death for any reason. Finally, we collected the post-progression information including the failure patterns and subsequent treatments. To understand the failure patterns of those patients, we defined “new” lesions of failure as tumors not existing before ICI treatment, whereas “existing” lesions were those existing sites that responded to treatment but then progressed. We also recorded the number of progression sites after ICI therapy. Oligo-progression refers to a clinical scenario in which progression sites were less than three (14).

### Statistical Analysis

PFS, TTP, and OS were calculated by the Kaplan–Meier method. Continuous and categorical variables were described using means and percentages, respectively. All the analyses were performed according to the Statistical Package for the Social Science (SPSS) version 24.0. Each test was performed at the 0.05 significance level and all *P*-values were two-sided. The graphs were performed by GraphPad Prism version 8.4.3.

## Results

### Baseline Patient Characteristics

The median follow-up time was 34.1 months (range, 2.8–53.17 months). One hundred and eighteen patients with clinical benefit to ICIs were identified. The characteristics of patients are described in **Table 1**. Among the 118 responders, 96 (81%) are male and ages ranged from 34 to 83 with a median age of 62 years. In terms of smoking status, 77 (65%) were current or ex-smokers and 41 (35%) patients never smoked. The smoking index of 62% of smokers was over 400 (smoking index = number of cigarettes smoked per day × years of smoking). Eighty (68%) patients had adenocarcinoma and 38 (32%) had non-adenocarcinoma NSCLC. Eleven (9%) patients had IIIB stage NSCLC, whereas the remaining 107 (91%) had IV NSCLC. Among them, 34 (29%) patients received first-line treatment, while 84 (71%) received second-line ICIs; 13.6% patients possessed high expression of PD-1/PD-L1 with tumor proportion score (TPS) ≥50%, which primarily received fist-line immunotherapy. It was only known that the status of PD-1/PD-L1 expression was positive among 11% patients and 75.4% remained unknown (**Figure S1**). When it comes to the ICI agent, 54 (45.8%) patients accepted nivolumab, 37 (31.4%) atezolizumab, 14 (11.9%) durvalumab, 9 (7.6%) pembrolizumab, and 4 received camrelizumab. Ninety-four (79.7%) patients adopted ICI monotherapy, and the rest accepted combination therapy, including chemotherapy (16.9%), anti-cytotoxic T-lymphocyte-associated protein-4 (anti-CTLA-4) therapy (1.7%), and antiangiogenic therapy (1.7%). Initial patterns of disease burden concerning location were also described (**Table S1**). The bone was the most common site harboring metastasis at presentation irrespective of failure pattern, followed by the lung, brain, liver, and adrenal.

**Table 1:** Characteristics of patients.

Characteristics	No. of patients (%) (*n* = 118)
**Age in years, median (range)**	62 (34–83)
**Gender**	
Male	96 (81%)
Female	22 (19%)
**Smoking status**	
Current or ex-smoker	77 (65%)
Never	41 (35%)
**Histology**	
Adenocarcinoma	80 (68%)
Non-adenocarcinoma	38 (32%)
**Disease stage**	
IIIB	11 (9%)
IV	107 (91%)
**Treatment line**	
First-line treatment	34 (29%)
Second-line treatment	84 (71%)
**Treatment**	
Nivolumab	54 (46%)
Atezolizumab	37 (31%)
Durvalumab	14 (12%)
Pembrolizumab	9 (8%)
Camrelizumab	4 (3%)
**Best objective response**	
Stable disease	78 (66%)
Partial response	40 (34%)
Complete response	0

### Survival Analysis

**Figure 1:**
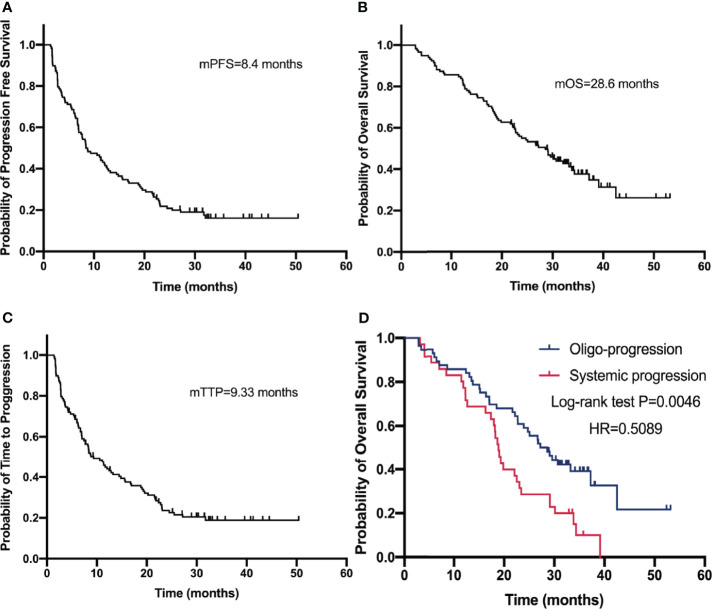
Progression-free survival **(A)**, overall survival **(B)**, and time to progression **(C)** curves of 118 patients. Overall survival **(D)** of oligo-progression patients and systemic progression patients.

Before the cutoff date, 72 (61%) patients had died. The median PFS (mPFS) was 8.40 months [95% confidence interval (CI), 5.15–11.65 months; **Figure 1A**] and the median OS (mOS) was 28.6 months (range, 23.00–34.20 months; **Figure 1B**). The median TTP (mTTP) was 9.33 months (range, 6.40–12.26 months; **Figure 1C**). The mOS of oligo-progression patients and systemic progression patients was 27.23 (range, 21.92–32.54) and 18.87 (range, 17.53–20.22) months, respectively (**Figure 1D**). The mOS of patients who received different post-progression treatment was also analyzed: the mOS of patients who received post-progression systemic treatment was 22.53 months, whereas those who received consolidative radiotherapy was 23.07 months. The OS and TTP among patients of TPS score ≥50% and all patients were depicted (**Figure S2**). The mOS and mTTP of patients possessing a high PD-L1 expression (≥50%) were 29.13 months and 8.3 months, respectively, which showed no statistical difference compared with the all-patient population.

To explore the relationship between ICI dosage and response, patients were divided into a higher-dose group and a lower-dose group according to the median of total ICI dosage that they received throughout the treatment. Of the population who received atezolizumab, the higher-dose group showed prolonged mOS (34.33 months, 95% CI 29.488–39.172 months) compared with the lower-dose group (22.53 months, 95% CI 16.089–28.971 months, *P* = 0.0012, HR = 0.2786). The same tendency was also found among the nivolumab group: 32.07 *vs.* 15.2 months (*P* = 0.04, HR = 0.5032, **Figure S3**).

### Response to ICI Therapy and Patterns of Failure Analysis

**Figure 2:**
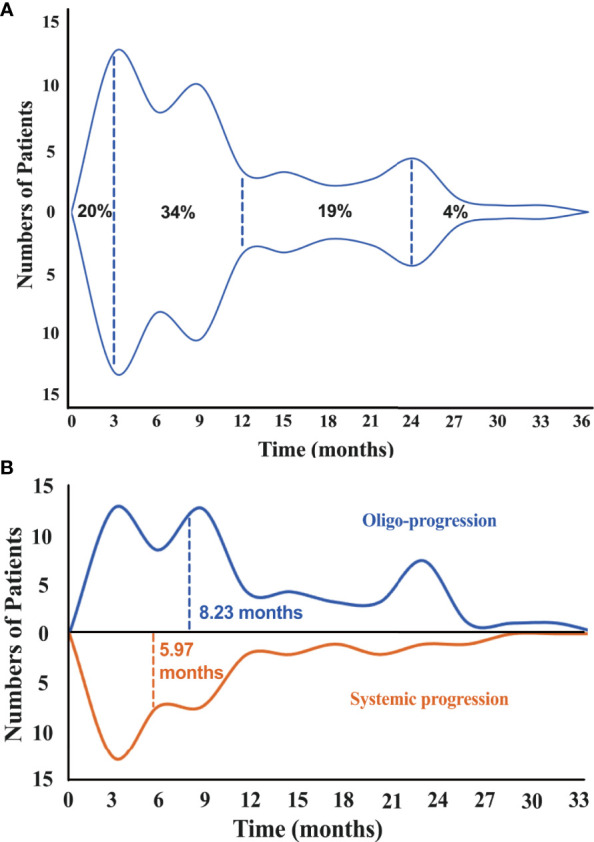
**(A)** Proportion of progressed patients varies over time. The area enclosed by the upper and lower curves represents the number of patients. **(B)** Median time of disease progression among patients with oligo-progression (above midline) and systemic progression (below midline).

**Figure 3:**
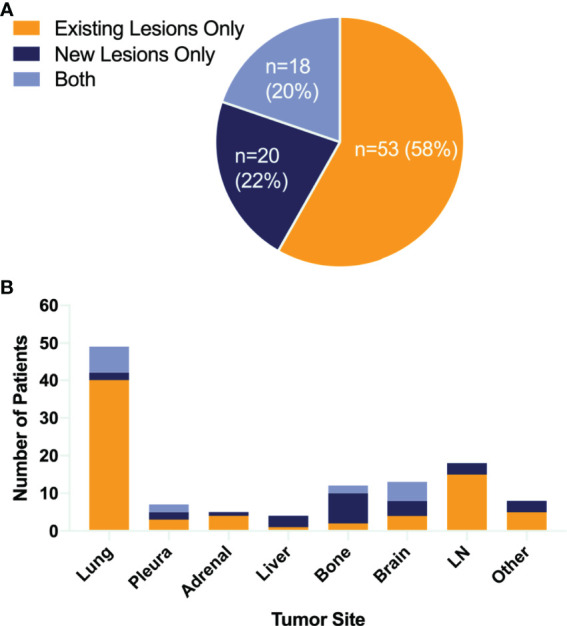
Patterns of failure. Pie chart **(A)** showing the proportion of different failure lesions. Bar graph **(B)** showing both sites of tumor growth and the number of patients. LN, lymph node.

In all 118 initial responders, none of the patients had CR, 40 (34%) patients had PR, while 78 (66%) subjects had SD. The progression characteristics of the patients are listed in **Table 2**. At the end of the follow-up interval, 91 (77.1%) patients experienced disease progression. The rate of disease progression varies over time as shown in **Figure 2**: 20% of patients progressed after 3 months of ICI treatment, 34% of patients progressed in the following 9 months, 19% of patients progressed in the second year of ICI treatment, and the remaining 4% of patients progressed after 2 years (**Figures 2A** and **S4**). The progression features were elaborately depicted (**Figure 3**). The vast majority of patients developed progression in existing lesions (*n* = 53, 58%), only 20 (22%) patients had progressed at new lesions, and 18 (20%) of them had both existing and new site progression. The most common lesion of progression was the lung, encountered in 49 (53.8%) patients. Among these patients, 40 subjects experienced progress in existing sites. Lymph nodes (LNs) also accounted for a considerable proportion (*n* = 18, 19.8%) and 15 progressed in existing sites. Following LNs, the percentage of progression in the brain and bone was about the same, with 13 and 12 patients, respectively. Then, the other sites of progression occurred in the pleura (*n* = 7, 7.7%), adrenal (*n* = 5, 5.5%), and liver (*n* = 4, 4.4%). There were four other patients having soft tissue invasion: three had pericardial effusion and one had intestinal metastasis.

**Table 2:** Progression characteristics.

Characteristics	No. of patients (%) (*n* = 91)
**Site of progression**	
New only	20 (22.0%)
Existing only	53 (58.2%)
Both	18 (19.8%)
**Number of progression lesions**	
≤2	56 (61.5%)
>2	35 (38.5%)
**Therapy type after progression**	
Systemic	59 (64.8%)
Radiation	6 (6.6%)
Systemic plus radiation	15 (16.5%)
None	11 (12.1%)
**Type of system therapy**	
Chemotherapy	64 (70.3%)
Antiangiogenic therapy	15 (16.5%)
Targeted therapy	6 (6.6%)
Immunotherapy	4 (4.4%)
**Alive**	
Yes	24 (26.4%)
No	67 (73.6%)

**Figure 4:**
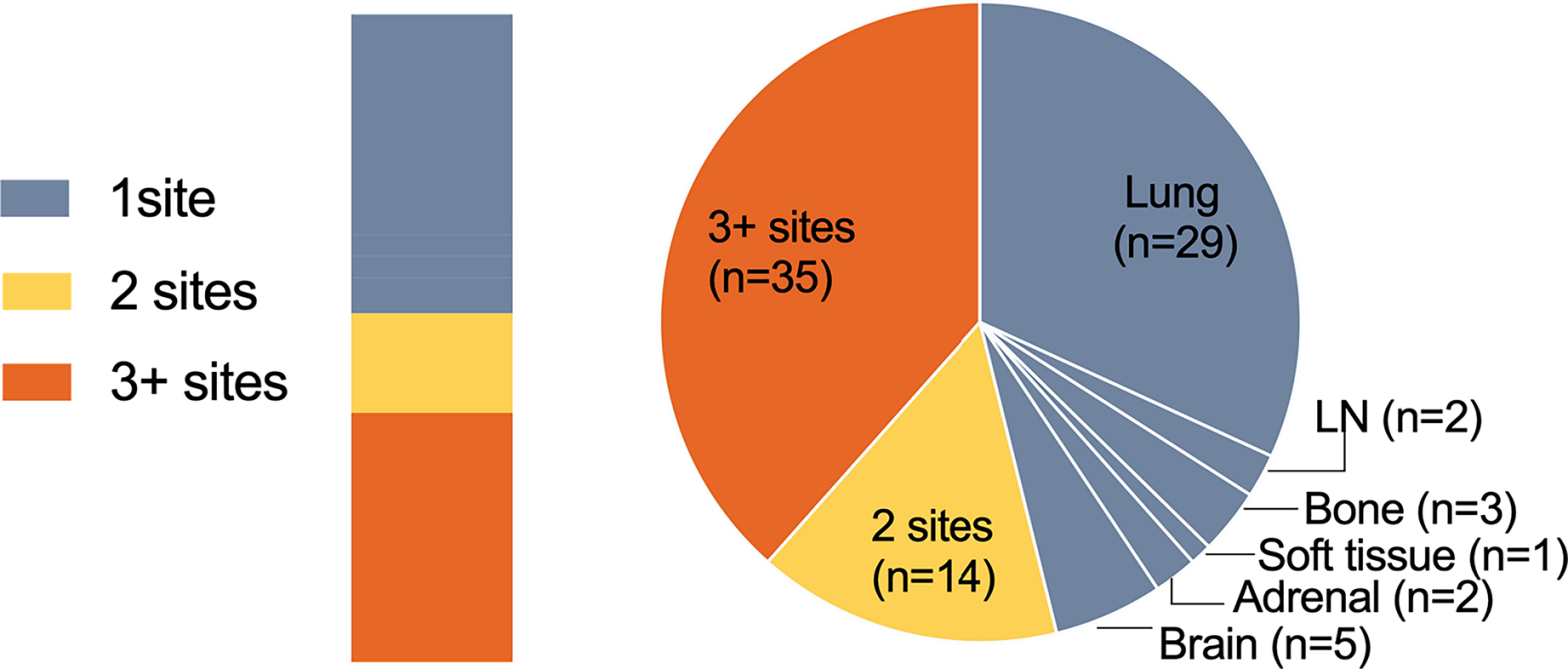
Numbers of progression sites. Pie chart showing the number of patients with one site (gray), two sites (yellow), or three or more sites (orange) of progressive disease.

As vividly shown in the pie chart (**Figure 4**), 56 (61.5%) patients showed ≤2 progression sites, 42 (46.2%) patients progressed in only one site, and 35 (38.5%) patients developed progression in more than two sites. For patients who experienced oligo-progression, the median time to disease progress was 8.23 months, while it was 5.97 months for those patients who had systemic progressed disease state (**Figure 2B**). The progression proportions of each organ are also revealed in **Figure 4**.

### Post-Progression Treatment

After progression, 74 patients received systemic treatment and 21 received radiation therapy, of which 6 patients received radiotherapy alone. Eight of the patients received lung radiation therapy, eight brain metastasis radiotherapy, four bone metastasis radiotherapy, one adrenal gland radiotherapy, and one chest wall radiotherapy. The remaining 11 did not receive further treatment. Of those who received post-progression systemic therapy, four patients continued immunotherapy. Other types of systemic therapy included chemotherapy (*n* = 64), antiangiogenic therapy (*n* = 15), and molecular targeted therapy (*n* = 6).

### Immune-Related Side Effects

We also collected the immune-related side effects. We observed that 88 (74%) patients had treatment-related adverse events, 11 (9.3%) experienced second-degree immune-related side effects, 4 (3.4%) had third-degree side effects, and 1 patient stopped immunotherapy due to immune thyroiditis. Specific immune-related side effects include immune-associated pneumonia (*n* = 22), of which the main symptoms were chest distress and shortness of breath; immune-related endocrine problems (*n* = 15), such as hyperthyroidism (*n* = 1)/hypothyroidism (*n* = 7), fatigue (*n* = 12), hair loss (*n* = 1), and constipation (*n* = 2); immune-associated hepatitis (*n* = 7), characterized by elevated liver enzymes (e.g., alanine transaminase/aspartate aminotransferase, ALT/AST); immune-related skin problems (*n* = 27), such as pruritus, rash, and macular papule; immune-associated enteritis (*n* = 2); and neurological problems (*n* = 7) mainly manifested as muscle numbness.

## Discussion

In our study, we found that the mPFS of patients in our cohort was 8.40 months and the mOS was 28.6 months, which was consistent with previous studies (3, 5). After 5 years of follow-up, 77% of patients had disease progression. A large proportion of patients (34%) progressed in the last 9 months of the first year of ICI treatment and 19% of patients progressed in the second year. Besides, the oligo-progression occurred later than systemic progression: the median time to progression of the former was 8.23 months, while the latter was 5.97 months. The oligo-progression patients showed prolonged mOS compared with patients with systemic progression.

In patterns-of-failure analysis, the vast majority of patients developed oligo-progression (≤2 sites), and 65% of them developed at only one site, which was consistent with previous failure patterns of chemotherapy and TKI therapy. Besides, nearly 60% enrolled patients progressed at “existing” sites. In terms of progression lesion, the most frequent lesions of progression were the lung and LNs. More than 50% of the patients had lung progression and 81.6% (40/49) of them were existing site failure. LNs also accounted for a considerable proportion of 19.8% and 83.3% (15/18) progressed in existing sites.

In the era of chemotherapy and EGFR-TKI targeting treatment, patterns-of-failure results are highly consistent with our ICI treatment outcome. For instance, the study of Rusthoven et al. uncovered that the predominant patterns of failure in patients with advanced NSCLC after first-line systemic chemotherapy were local only (15). Al-Halabi et al. conducted the first study to report a detailed analysis of the patterns of disease progression after TKI therapy in patients with metastatic EGFR-mutant NSCLC, and they indicated that failure in residual sites of original disease was likely to occur before the development of new sites of distant metastasis (11). For these patients who had oligo-progression, previous studies have also shown that the combination of local consolidation radiotherapy may markedly improve clinical efficacy (16, 17). A phase 2 randomized clinical trial (NCT02045446) indicated that consolidative stereotactic ablative radiotherapy (SABR) showed nearly tripling PFS compared with maintenance chemotherapy alone in patients with limited metastatic NSCLC after achieving a PR/SD after first-line chemotherapy (18). Another phase 2 clinical trial (NCT01725165) came to a similar conclusion, and the authors found that the addition of local consolidative therapy delayed the appearance of new lesions, suggesting that the advantage of consolidative radiotherapy could extend beyond known sites of disease (19). Based on the above evidence and our results, in patients with advanced NSCLC who received ICI therapy, local consolidation therapy (surgery, radiotherapy, or thermal ablation) should be considered. The lung and LNs may be particularly susceptible sites to acquired resistance. We wonder if there is a possibility to consolidate aggressive consolidative radiotherapy at the point of maximal response during or immediately after immunotherapy for advanced NSCLC.

As a retrospective analysis, our study has some limitations. Firstly, patients engaged in the study were screened out from a single center, resulting in a small number of patients. The gender distribution in the enrolled population was uneven, with 81% of the patients being male. Secondly, the number of first-line ICI-treated patients was relatively small, and there were eight patients expressing EGFR gene mutations in the population. According to previous clinical research experiences, these patients should be given priority to molecular targeted therapy (20, 21). Apart from this, the types of ICI reagents were complicated. Finally, since most patients received second-line treatment, the PD-L1 expression level was not obtained before enrollment, which is an important clinical indicator to predict the effect of ICI immunotherapy (22).

## Conclusions

In this study, we revealed that the majority of NSCLC patients progressed in pre-existing lesions after ICI immunotherapy, and the progression tended to be oligo-progression. This group of patients may benefit from the adoption of local therapy. The participation of local therapy, such as consolidative radiotherapy, at the point of maximal response during or immediately after systemic ICI immunotherapy for advanced NSCLC, may delay the progression of this part of the patients and improve their survival outcome. It is hoped that our research will throw light on future prospective research to explore multiple combination treatment with ICI and its specific participation time considering the balance of efficacy and safety.

## Data Availability Statement

The raw data supporting the conclusions of this article will be made available by the authors, without undue reservation.

## Ethics Statement

The studies involving human participants were reviewed and approved by Shanghai Chest Hospital. The patients/participants provided their written informed consent to participate in this study.

## Author Contributions

QZ, XC, and RC contributed to the conception and design of the study. RC, YY, and QZ participated in the provision of study materials or patients. RC and YY contributed to the collection and assembly of data. XF provided administrative support. All authors contributed to the article and approved the submitted version.

## Funding

This work was supported by the National Natural Science Foundation of China (No. 81703021 to QZ) and Shanghai Pujiang Program (No. 2020PJD56 to QZ).

## Conflict of Interest

The authors declare that the research was conducted in the absence of any commercial or financial relationships that could be construed as a potential conflict of interest.

## Publisher’s Note

All claims expressed in this article are solely those of the authors and do not necessarily represent those of their affiliated organizations, or those of the publisher, the editors and the reviewers. Any product that may be evaluated in this article, or claim that may be made by its manufacturer, is not guaranteed or endorsed by the publisher.
