# Supplementary material for: Patterns of Failure in Patients With Advanced Non-Small Cell Lung Cancer Treated With Immune Checkpoint Inhibitors
Source: Front Oncol. 2021 Sep 7;11:724722. doi: 10.3389/fonc.2021.724722 (PMC8454403; doi:10.3389/fonc.2021.724722)
Supplement: Supplementary file 1 [file DataSheet_1.docx]

Supplementary Material

# Supplementary Figures


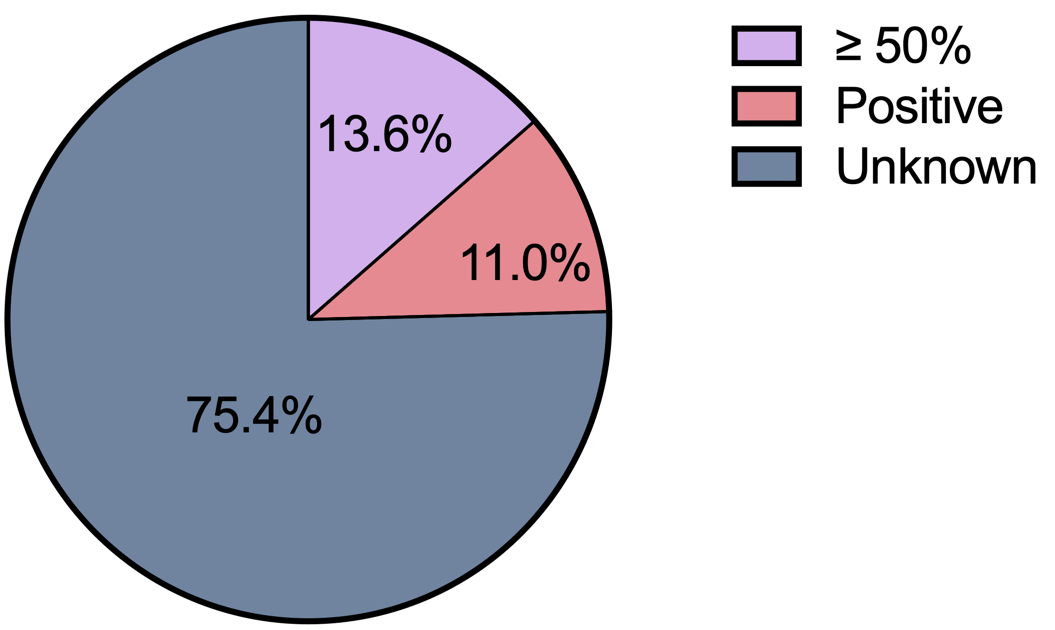


**Figure S1.** TPS score of patients (n=118). 13.6% patients possessed high expression of PD-1/PD-L1 with TPS score of ≥50%, which primarily received fist-line immunotherapy. It is only known that the status of PD-1/PD-L1 expression were positive among another 11% patients and 75.4% remained unknown.


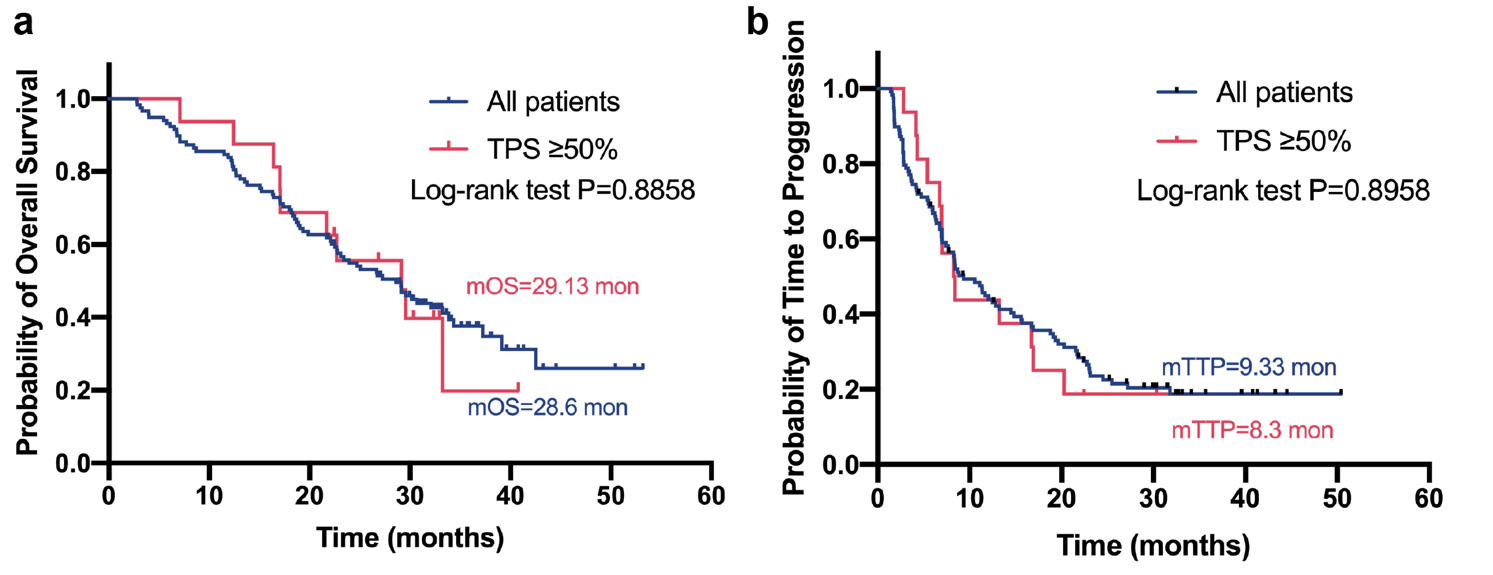


**Figure S2.** Overall Survival (OS) and time to progression (TTP) among patients of TPS score ≥50% and all patients.

**Figure S3.** Relationship between dosage and survival among patients receiving Atezolizumab and Nivolumab.


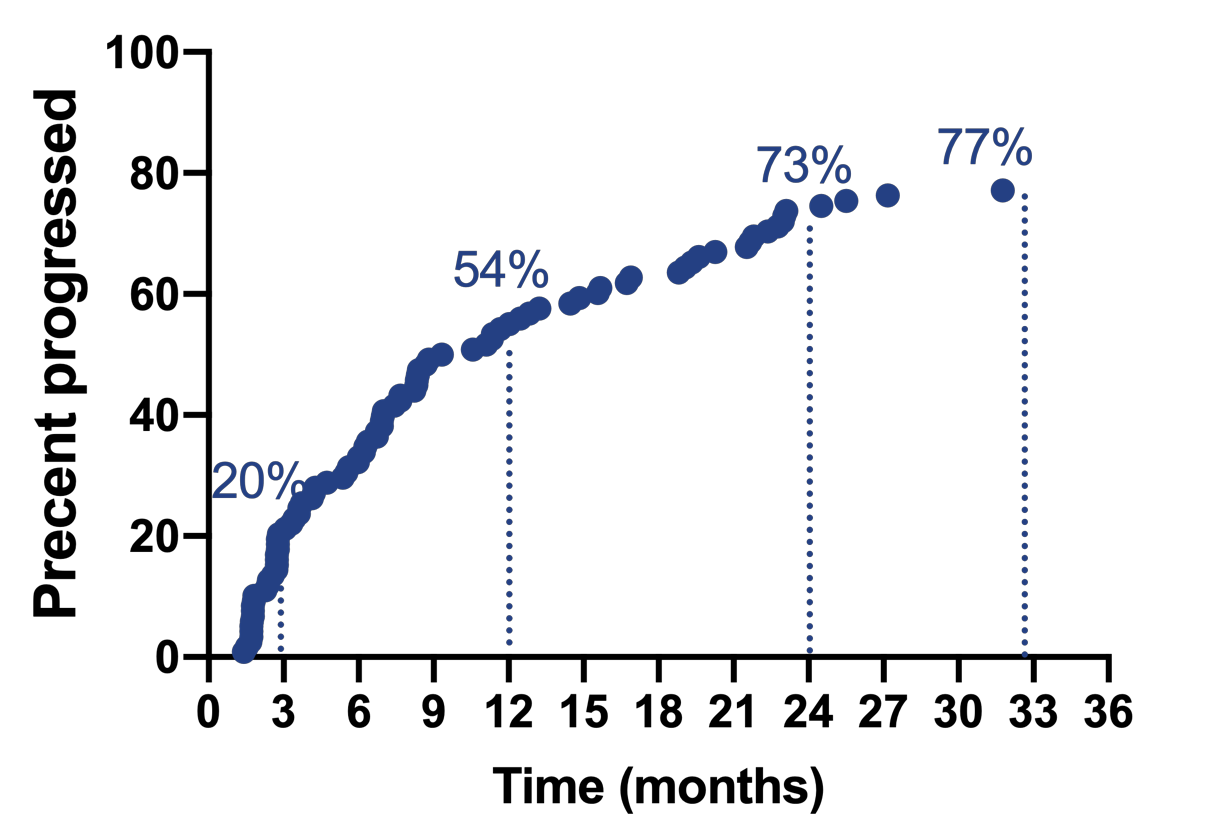


**Figure S4. Disease progressed precent varies over time.** 77% patients with initial clinical benefit developed disease progression during follow up interval.

**Table S1. Initial Characteristics of disease burden**

| Variable | n (n=118) | | % |
| --- | --- | --- | --- |
| T stage | |  |  |
| T0 | | 9 | 7.6 |
| T1 | | 12 | 10.2 |
| T2 | | 49 | 41.5 |
| T3 | | 10 | 8.5 |
| T4 | | 38 | 32.2 |
| N stage | |  |  |
| N0 | | 10 | 8.5 |
| N1 | | 4 | 3.4 |
| N2 | | 44 | 37.3 |
| N3 | | 60 | 50.8 |
| Organs harboring metastases | |  |  |
| Lung | |  |  |
| Yes | | 39 | 33.1 |
| No | | 79 | 66.9 |
| Brain | |  |  |
| Yes | | 25 | 21.2 |
| No | | 93 | 78.8 |
| Liver | |  |  |
| Yes | | 10 | 8.5 |
| No | | 108 | 91.5 |
| Adrenal | |  |  |
| Yes | | 10 | 8.5 |
| No | | 108 | 91.5 |
| Bone | |  |  |
| Yes | | 42 | 35.6 |
| No | | 76 | 64.4 |
| Other | |  |  |
| Yes | | 35 | 29.7 |
| No | | 83 | 70.3 |
| Number of organs of metastases | |  |  |
| 1 | | 55 | 46.6 |
| 2 | | 35 | 29.7 |
| 3 | | 20 | 16.9 |
| 4 | | 5 | 4.2 |
| $\geq$5 | | 3 | 2.5 |
| Number of metastases | |  |  |
| 1-5 | | 48 | 40.7 |
| 6-10 | | 46 | 39.0 |
| >10 | | 24 | 20.3 |
